# Significant adverse prognostic events in patients with urosepsis: a machine learning based model development and validation study

**DOI:** 10.3389/fcimb.2025.1623109

**Published:** 2025-08-08

**Authors:** Yiqu Wei, Wanqing Xu, Shuo Yang, Congfeng Zhang, Jia Wang, Xianyao Wan

**Affiliations:** ^1^ Department of Critical Care Medicine, The First Affiliated Hospital of Dalian Medical University, Dalian, China; ^2^ Department of Critical Care Medicine, Dandong Central Hospital, Dandong, China; ^3^ Department of Oncology, The First Affiliated Hospital of Zhengzhou University, Zhengzhou, China; ^4^ Department of Anesthesiology, The Second Affiliated Hospital of Dalian Medical University, Dalian, China

**Keywords:** machine learning, urosepsis, prognostic model, MIMIC-IV database, SHAP

## Abstract

**Background:**

Urosepsis is a subset of sepsis with a high mortality rate. Currently, the ranking of urosepsis in sepsis etiology is on the rise. Our goal is to use machine learning (ML) methods to construct and validate an interpretable prognosis prediction model for patients with urosepsis.

**Method:**

Data were collected from the Intensive Care Medical Information Mart IV database version 3.1 and divided into a training cohort and a validation cohort in a 7:3 ratio. Random Forest (RF), Lasso, Boruta, and eXtreme Gradient Boosting (XGBoost) were used to identify the most influential variables in the model development dataset, and the optimal variables were selected based on achieving the λ_1se_ value. Model development includes seven machine learning methods and ten cross validations. Accuracy and Decision Curve Analysis (DCA) were used to evaluate the performance of the model in order to select the optimal model. Internal validation of the model included area under the ROC curve (AUC), sensitivity, specificity, Matthews correlation coefficient, and F1-score. Finally, SHapley Additive exPlans (SHAP) was used to explain ML models.

**Result:**

A total of 1389 patients with urosepsis were included. Optimal predictors were selected through statistical regularization, yielding a parsimonious set of 9 variables for model development. The performance of XGBoost model is the best and the accuracy of XGBoost was 0.818, with an AUC of 0.904 (95% CI: 0.886-0.923). The internal validation accuracy was 0.797, AUC was 0.869 (95% CI: 0.834-0.904), sensitivity was 0.797, specificity was 0.752, Matthews correlation coefficient was 0.597, and F1-score was 0.791. This indicates that the predictive model performs well in internal validation. SHAP-based summary graphs and diagrams were used to globally explain the XGBoost model.

**Conclusion:**

ML demonstrates strong prognostic capability in urosepsis, with the SHAP method providing clinically intuitive explanations of model predictions. This enables clinicians to identify critical prognostic factors and personalize treatments. While our model achieved high predictive accuracy, its retrospective derivation from a single-center database necessitates external validation in diverse populations, which should be addressed through future prospective multicenter studies to establish clinical generalizability.

## 1 Introduction

Urosepsis is caused by infection of the genitourinary system, which accounts for about 9% to 31% of sepsis cases, and is one of the worst prognosis diseases for patients with urinary tract infections (Porat et al., 2025). For certain specific populations, the case fatality rate of urosepsis is approximately 25%-60%, highlighting the significant clinical importance of improving the early diagnosis and management of urosepsis (Li et al., 2024a). Considering the high incidence rate and mortality of urosepsis, it is necessary to establish a reliable and effective prognosis model.

Several risk prediction models for urosepsis patients have been widely studied and established. Villanueva-Congote (Villanueva-Congote et al., 2024) et al. have shown that the Neutrophil-to-Lymphocyte Ratio (NLR) and Platelet-to-Lymphocyte Ratio (PLR) may be valuable prognostic indicators for predicting the risk of urosepsis. They can help clinicians with early risk stratification, timely intervention, and resource allocation. Croghan (Croghan et al., 2023) et al. conducted a prospective multi-institutional study, recording the baseline and continuous ureteroscopic intrarenal pressure (IRP) of patients during ureteroscopy (URS) surgery, and found that there seemed to be a relationship between elevated IRP and postoperative urosepsis. Canat (Canat et al., 2020) et al. found that the elevated level of procalcitonin (PCT) on the second day after prostate biopsy was a statistically significant independent predictor of urosepsis. It can be used as an early biomarker to predict the occurrence of urosepsis after prostate biopsy. However, the above predictive models are based on traditional COX regression that suffers from limitations including its reliance on the proportional hazards assumption, inability to estimate baseline hazard functions directly, sensitivity to multicollinearity, and inadequate handling of high-dimensional data and nonlinear relationships, necessitating more advanced methods for complex datasets.

In recent years, various machine learning (ML) algorithms, a method of data analysis that develops algorithms to predict outcomes by learning from data, have been studied for the early detection of urosepsis. It is superior to traditional statistical methods and does not require assumptions about input variables and their relationship with outputs. The advantage of fully data-driven learning without relying on rule-based programming is that ML constitutes a reasonable approach. Researchers have successfully applied a variety of ML paradigms to improve the generalization ability of models in complex clinical scenarios, such as Gradient Boosting Decision Trees (GBDT), Random Forests (RF) and Deep Neural Networks (DNN). These methods further enhance the interpretability of the model through the pathophysiological associations revealed by feature importance analysis (Su et al., 2023). The occurrence of urosepsis after PCNL surgery is one of the main reasons for the increased mortality. Li (Li et al., 2024b) et al. collected important preoperative and intraoperative clinical data of patients and established a model combined with ML methods that for predicting the occurrence of urosepsis after PCNL. The results showed that the model had a good predictive effect on the occurrence of urosepsis after PCNL (AUC = 0.89). In addition, they also found that the change of platelet counts before and after surgery was an important predictive factor (Li et al., 2024b). However, although ML algorithms have performed well in previous studies, due to the “black box” nature of ML algorithms, it is difficult to interpret which characteristics of patients are responsible for a given prediction. In addition, the main result of the above study is to detect the occurrence of urosepsis, rather than adverse clinical outcomes. Moreover, the sample size included in the study is too small, resulting in low clinical credibility.

Therefore, we use large-scale data based on MIMIC-IV database, to develop a prognosis prediction model for critically ill patients with urosepsis to improve the reliability of research conclusions. Our feature selection ensemble—incorporating Random Forest, Lasso, Boruta, and XGBoost—mitigates algorithm selection bias by spanning diverse learning paradigms, thereby generating robust variable rankings essential for clinical modeling. In addition, to explain the results of the ML model, we combine advanced ML algorithms based on SHapley Additive exPlans (SHAP), a popular ML technique for a deeper understanding of the complex relationship between features and predictions. In addition to optimizing the predictive performance of mortality risk in critically ill patients with urosepsis, this study also provides intuitive explanations that will help clinicians fully understand how the developed model makes specific predictions and increase opportunities for early intervention.

## 2 Methods

### 2.1 Source of data

An open and free intensive care database called Medical Information Mart for Intensive Care IV (MIMIC-IV) version 3.1 (Johnson et al., 2023; Wang et al., 2024b), which contains the latest version of comprehensive clinical data of patients admitted to Beth Israel Deaconess Medical Center in Massachusetts from 2008 to 2022. MIMIC-IV contains data on 65000 patients admitted to the ICU and 200000 patients admitted to the emergency room. The clinical data in the database include demographic characteristics, vital signs, imaging examinations, laboratory test results, data dictionaries and documents containing the codes of the ninth and tenth editions of the international classification of diseases (ICD-9 and ICD-10, respectively), as well as hourly physiological data records beside the monitors verified by ICU nurses. The health information obtained from the MIMIC-IV database could not be identified, so the informed consent of patients was not required (Goldberger et al., 2000; Oweira et al., 2018). This study was approved to extract data from the database for research purposes (certification number: 58407754). The database has been approved by the Massachusetts Institute of Technology (MIT) institutional review board (IRB).

### 2.2 Study design and population

**Figure 1:**
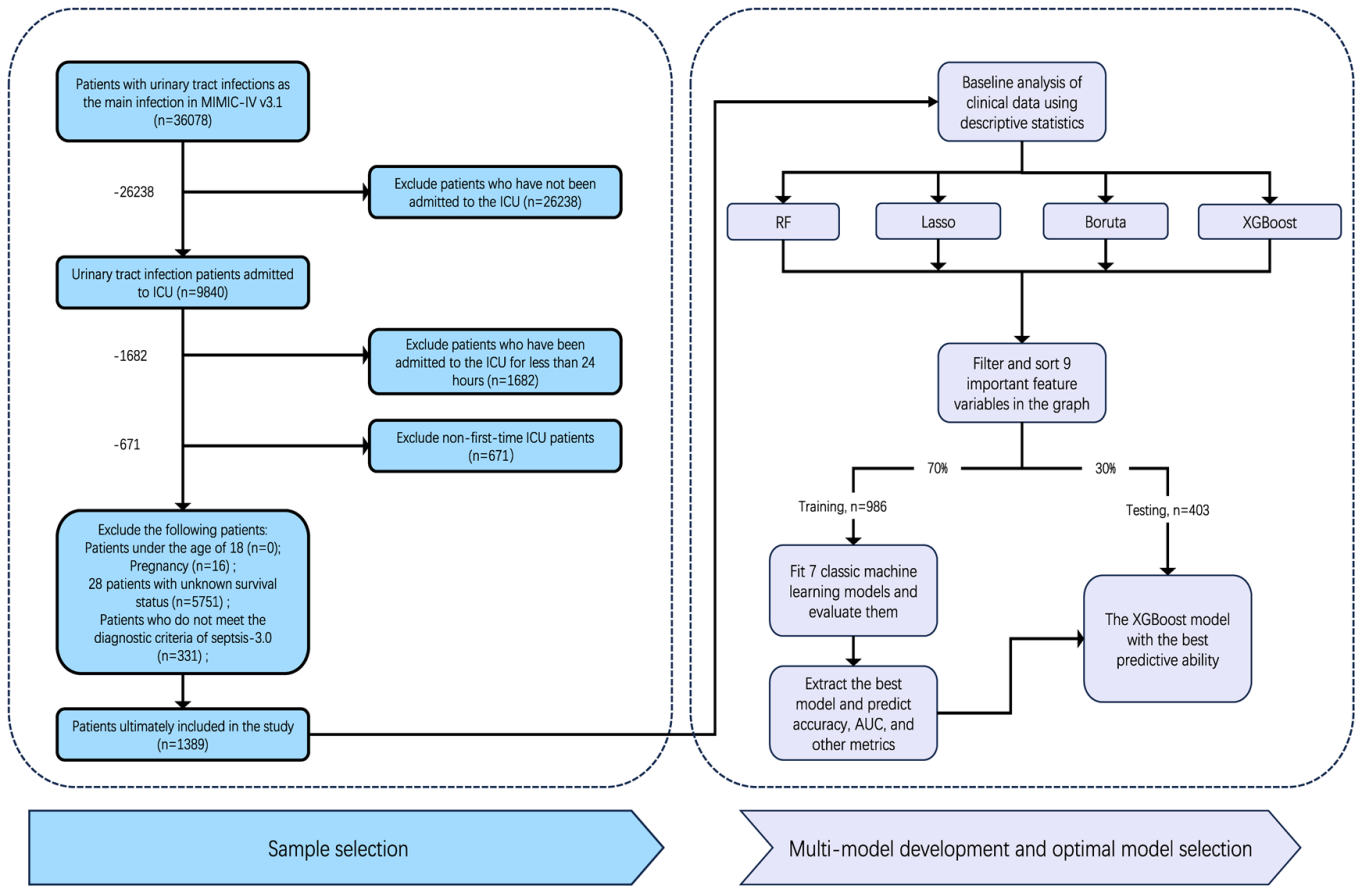
Study cohort selection and model development workflow. From 58,078 urinary tract infection patients in MIMIC-IV, 1,389 met inclusion criteria. Twelve key features were selected using four machine learning methods (RF, Lasso, Boruta, XGBoost), refined to nine variables through clinical review. The cohort was split 7:3 (training: testing). Seven ML models were trained; XGBoost demonstrated optimal performance and was validated.

The study focused on patients with urosepsis who were subsequently hospitalized and admitted to the ICU for the first time. According to the definition of Sepsis-3.0, sepsis is defined as life-threatening organ dysfunction caused by a dysregulated host response to infection (Ohbe et al., 2024). Organ dysfunction represents at least two points identified as acute and infection-related changes in the Sequential Organ Failure Assessment (SOFA). Urosepsis is sepsis originating from the urogenital tract. The diagnosis of urosepsis requires both suspicion of sepsis as well as evidence of a urinary tract infections (UTI). In this study, we included common types of urinary tract infections, including pyelonephritis, cystitis, and urinary tract infections. The codes in ICD-9 and ICD-10 include 590, 595, 599.0 and N30. Patients who met the following criteria in the database were selected for this study: [1] first admission to ICU; [2] ICU stay >24 hours; [3] Age >18 years old; [4] It meets the diagnostic criteria of sepsis 3.0; [5] There is conclusive evidence of urinary tract infection, such as positive urine culture. The diagnosis code contains the diagnosis related to urinary tract infection and has a higher priority than other infections (Griebling, 2019). In the MIMIC-IV database, ICD-9 (99591, 99592, and 78552) and ICD-10 (R65.20, R65.21) codes were used to identify patients with sepsis. Following these criteria, we screened 1389 patients for the study (**Figure 1**). The final patient cohort was allocated to training cohort and validation cohort according to the ratio of 7:3 through stratified random partitioning for model establishment.

### 2.3 Data extraction

We first obtained the raw data using the Structured Query language of Navicat Premium software (version 16.3.8), including sociodemographic characteristics, vital signs, laboratory parameters, complications, and microbial information (**Supplementary Figure S1**) (Yang et al., 2020). We extracted the following demographic data: age, sex, race, weight, height, admission route, length of stay in the ICU, and hospital expiration flag (records of in-hospital deaths in the database) at the time of first admission to the ICU. Next, the vital signs of patients in the first 24 hours of ICU stay were collected, including mean arterial pressure (MAP), heart rate (HR), body temperature (T), respiratory rate (RR), saturation of peripheral oxygen (SpO2), urine volume, and then the laboratory parameters in the first 24 hours were collected, including blood routine examination, liver and kidney function, blood glucose, and arterial blood gas (ABG). In addition, advanced life support records such as mechanical ventilation and renal replacement therapy were also recorded. We removed more than 20% of the variables with missing observations, such as height and serum albumin level, to promote and ensure the accuracy of the study. Then, we used the mice and VIM package to process the missing data. Missing data were completely random. With the help of the RF algorithm, we performed 5 imputations of 50 iterations for the original missing data and completed the sensitivity analysis. When combining the characteristics of vital signs and relevant laboratory parameters, the maximum, minimum, and average values were used and considered as independent characteristics to be included in the study.

### 2.4 Clinical outcomes

The clinical outcome of the current study was 28 days All-Cause Mortality (ACM). Crucially, the time of death were specified as occurrences of death within a defined period following admission to the ICU, rather than merely identifying whether the patient was deceased at a specific time point.

### 2.5 Statistical analyses

Shapiro Wilks test was used for the normality test. Continuous variables with normal distribution were expressed as mean (SD, standard deviation) and compared with independent samples by T-test. Non normally distributed variables were expressed as median (interquartile range) and compared with the Kruskal Wallis test. Categorical variables were described as percentages and compared using the Chi-Square test.

**Figure 2:**
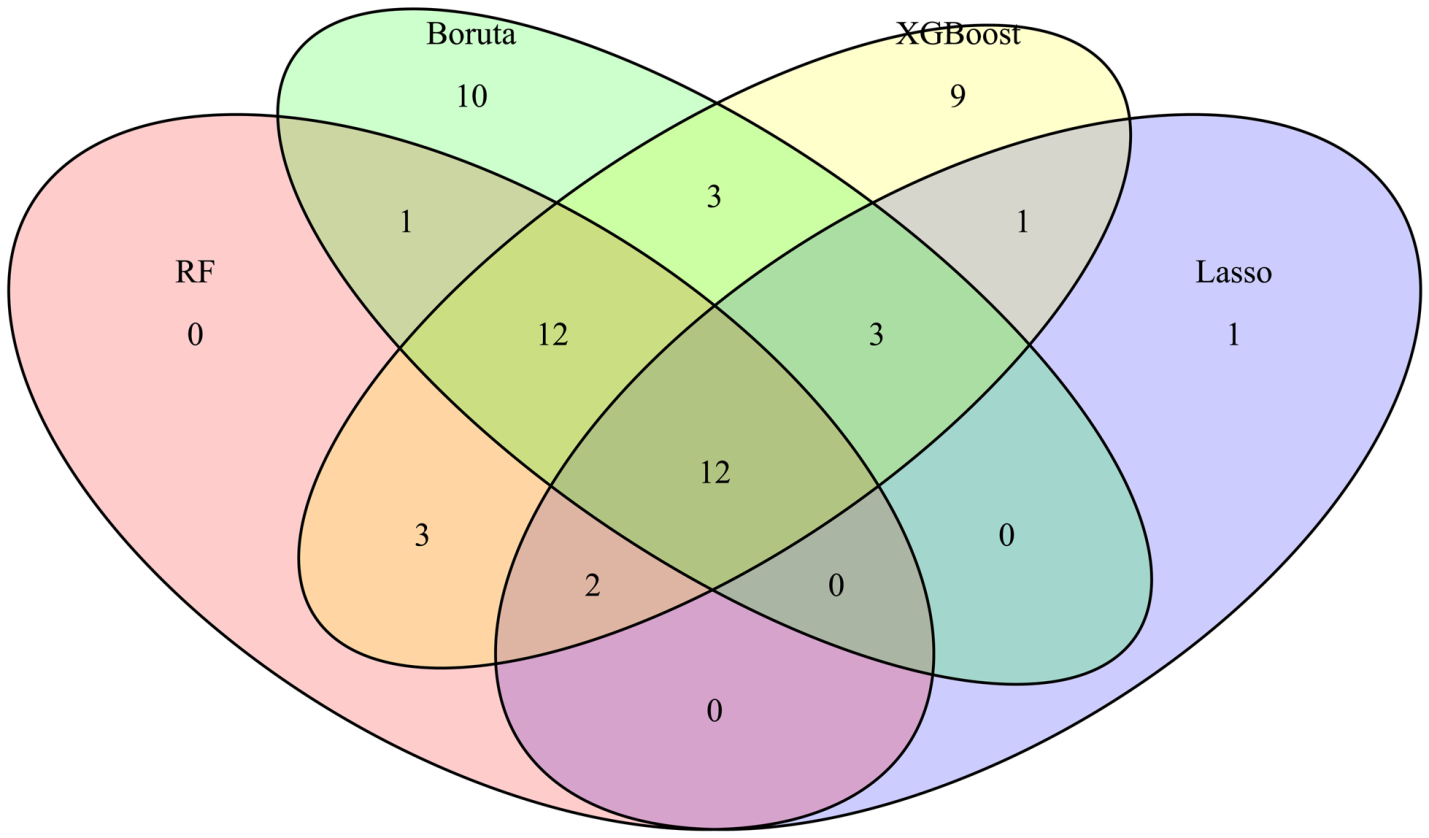
Variable Wayne diagram screened by four methods. The important variables and their intersection relationships selected by four feature selection methods (Boruta, XGBoost, Random Forest (RF), and Lasso) are presented in the form of Venn diagrams. The different colored blocks in the figure represent different methods and the intersection of color blocks represents the important variables selected by different methods together.

In addition, candidate data variables were additionally screened according to the principle of variable reduction to determine whether they were included in the model (**Figure 2**) (Venkatesh et al., 2023). RF is a classification algorithm composed of multiple decision trees. It constructs a machine learning model by randomly sampling training data and searching for the optimal segmentation solution. Each decision tree in the RF was constructed using feature measures aligned to dataset attributes (Farhadian et al., 2020; Shi et al., 2023), effectively evaluating the importance of each feature (Nachouki et al., 2023). Lasso can select variables through a series of parameters and reduce the complexity of the model, thus avoiding overfitting. Lasso’s complexity is controlled by λ, which eventually leads to a model with fewer variables. Compared with the traditional feature selection algorithm, Boruta is a packaging-based method to select features. Its goal is to identify the feature set with the greatest correlation with the dependent variable, rather than focusing solely on creating an optimized compact subset for a particular model (Zhou et al., 2023). By iteratively eliminating low correlation features, it effectively reduces signal noise and produces consistent classification performance (Sun et al., 2022b). At the same time, XGBoost stands out as an influential ensemble learning technology rooted in the classification tree framework; It combines low-accuracy classifiers into high-accuracy classifiers through iterative computation. The resulting ensemble classifier forms a decision tree interconnected by branches, which is a robust tool for effective classification (Moore and Bell, 2022). Specifically, we use RF, Lasso, Boruta, and XGBoost to model the variables of influencing factors. Then, we rank the variables with non-zero coefficients according to their impact on the outcome variables and identify common variables by taking the intersection of variables selected by all four methods. It should be noted that the complexity of the Lasso model is governed by the regularization parameter λ, where a 10-fold cross-validation procedure was implemented to determine the optimal λ values (λ_min_ or λ_1se_). Predictor selection was based on the minimum mean squared error criterion, as demonstrated in **Supplementary Figures S2** and **S3** (Kang et al., 2021; Wang et al., 2023).

## 3 Results

### 3.1 Baseline characteristics

A total of 1389 patients with urosepsis were included in this study, including 986 cases in the training cohort and 403 cases in the internal validation cohort. According to the survival status of patients within 28 days, patients were divided into the “Survival” group and the “Non-survival” group. In **Supplementary Table S1**, variables were shown and compared in groups of 28 days. In the training cohort, the 28-day ACM of patients with urosepsis was 48.07% (n = 986). The internal validation cohort demonstrated a 28-day ACM of 51.12%, representing a statistically significant 3.05 percentage point increase compared to the training cohort. In univariate analysis, significant differences were observed between the two groups in terms of age at admission, admission type, comorbidities such as heart failure, vital signs including SpO_2_ and MAP, laboratory parameters including Hct min, WBC min, Aniongap min, Aniongap max, Bicarbonate min, Urea Nitrogen min, Urea Nitrogen max, Potassium max, INR min, INR max, Pt min, Pt max, Ptt min, as well as scores such as SAPSII max, OASIS, and LODS max. Additionally, indicators such as Plt max, Bicarbonate max, Creatinine min, and Ptt max also demonstrated statistical significance between the two groups.

### 3.2 Features selected in models

**Figure 3:**
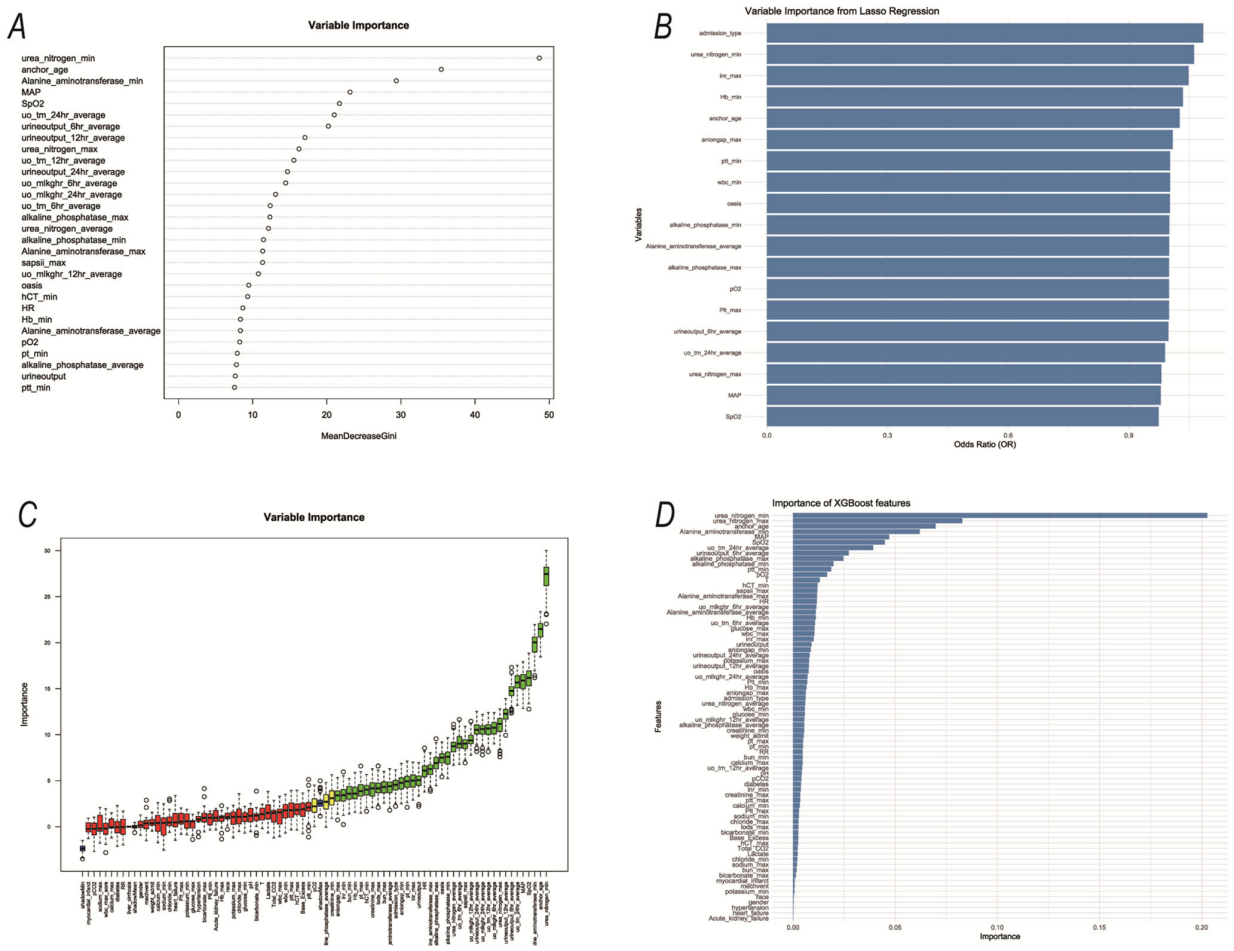
The variables selected by the four methods were sorted by importance. **(A)** RF; **(B)** Lasso; **(C)** Boruta; **(D)** XGBoost;.

Specifically, we use RF, Lasso, Boruta, and XGBoost to model the variables of influencing factors. Subsequently, we ranked the variables with non-zero coefficients according to their impact on the outcome variables and identified important variables by taking the intersection of variables selected by all four methods. The importance ranking of variables in the intersection set was shown in **Figure 3**. Finally, there are 9 variables used as predictive indicators, including urea nitrogen minimum, age, urine output (24-hour average), urine output (6-hour average), alkaline phosphatase maximum, SpO2, alkaline phosphatase minimum, MAP, and OASIS.

### 3.3 Model comparison

**Figure 4:**
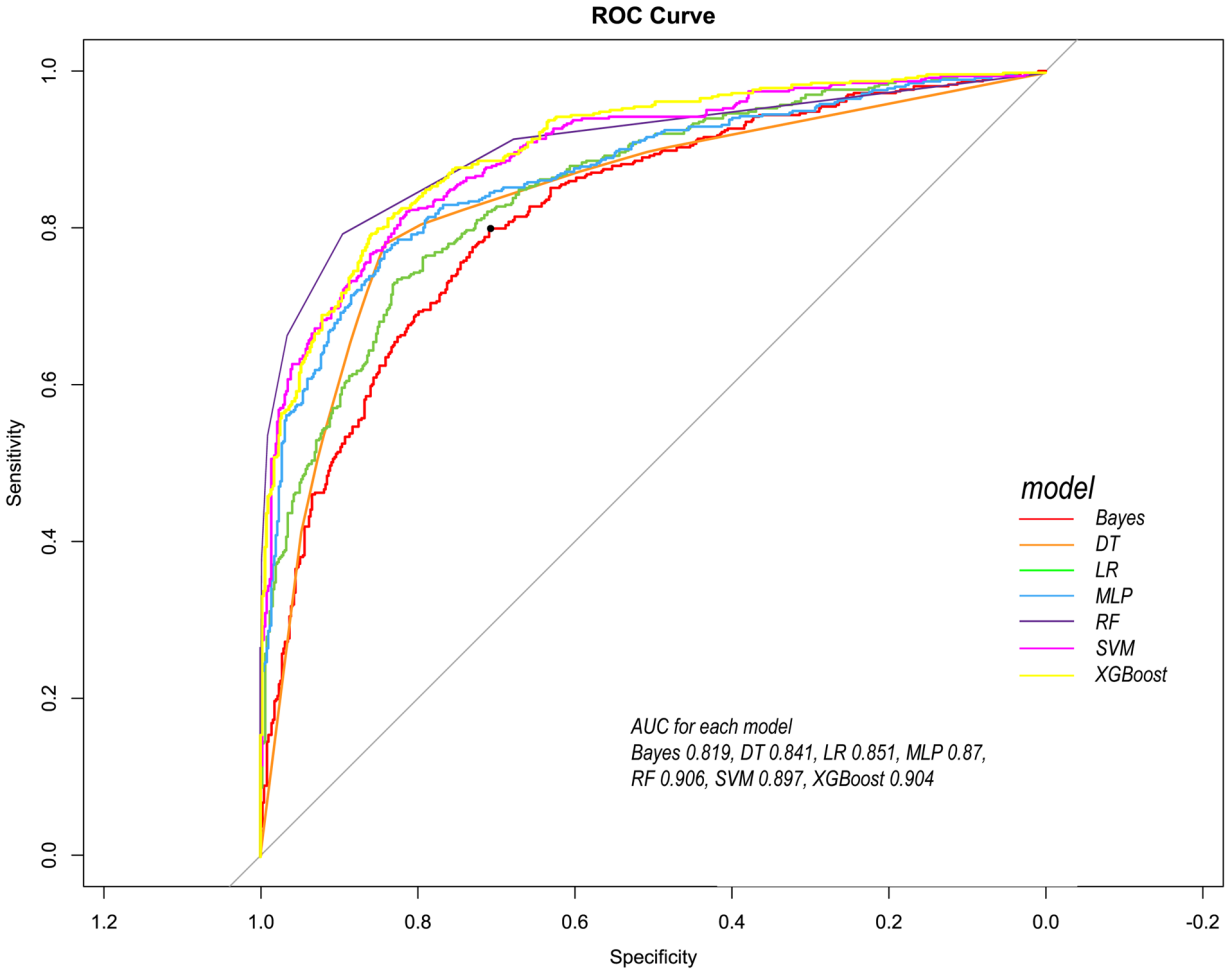
Comparison of ROC curves of seven models in the training cohort. Red line =Bayes model, orange line = DT model, green line = LR model, blue line = MLP model, dark purple line = RF model, bright purple line = SVM model, yellow line = XGBoost model.

**Figure 5:**
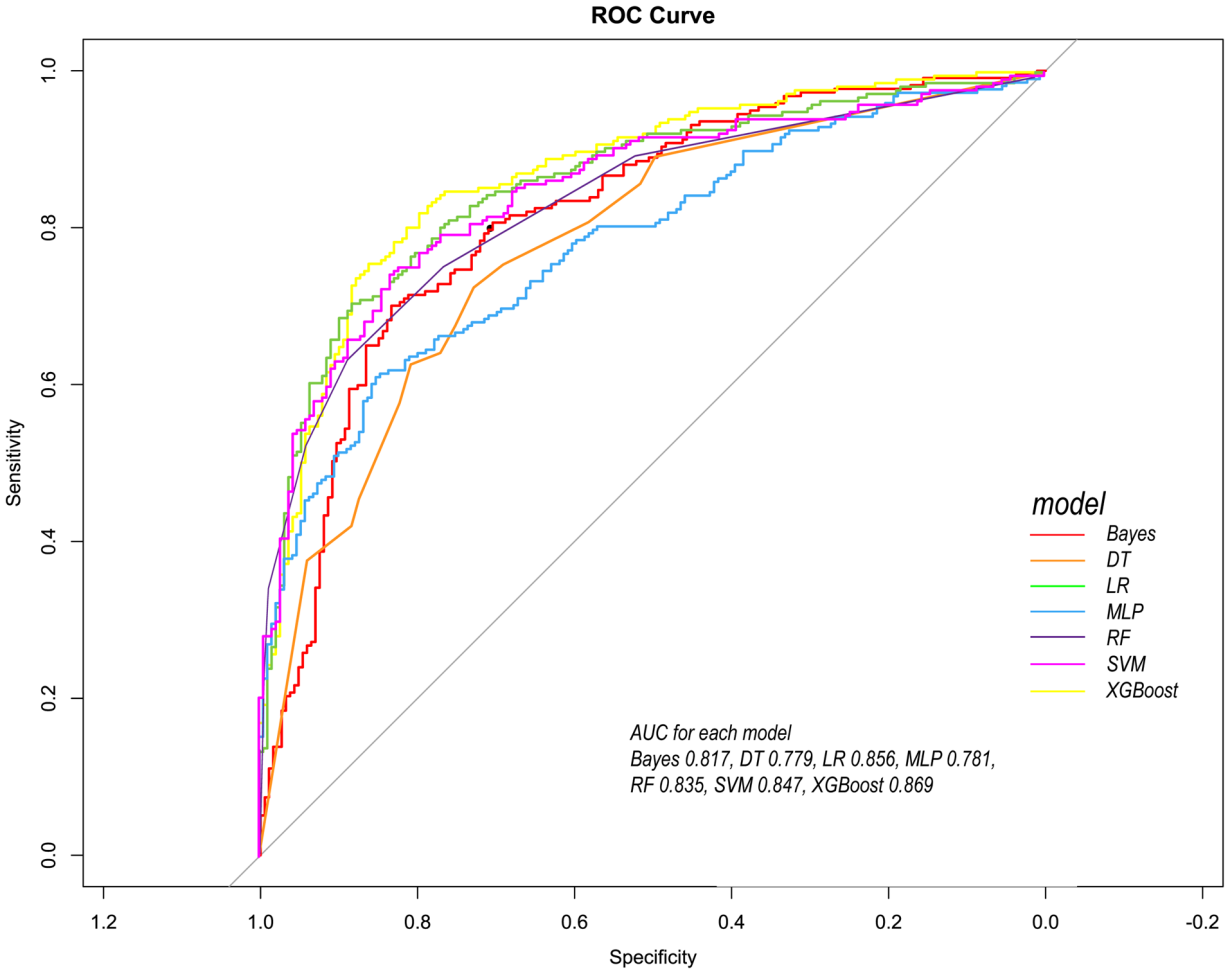
Comparison of ROC curves of seven models in the internal validation cohort. Red line = Bayes model, orange line = DT model, green line =LR model, blue line =MLP model, dark purple line =RF model, bright purple line = SVM model, yellow line = XGBoost model.

**Figure 6:**
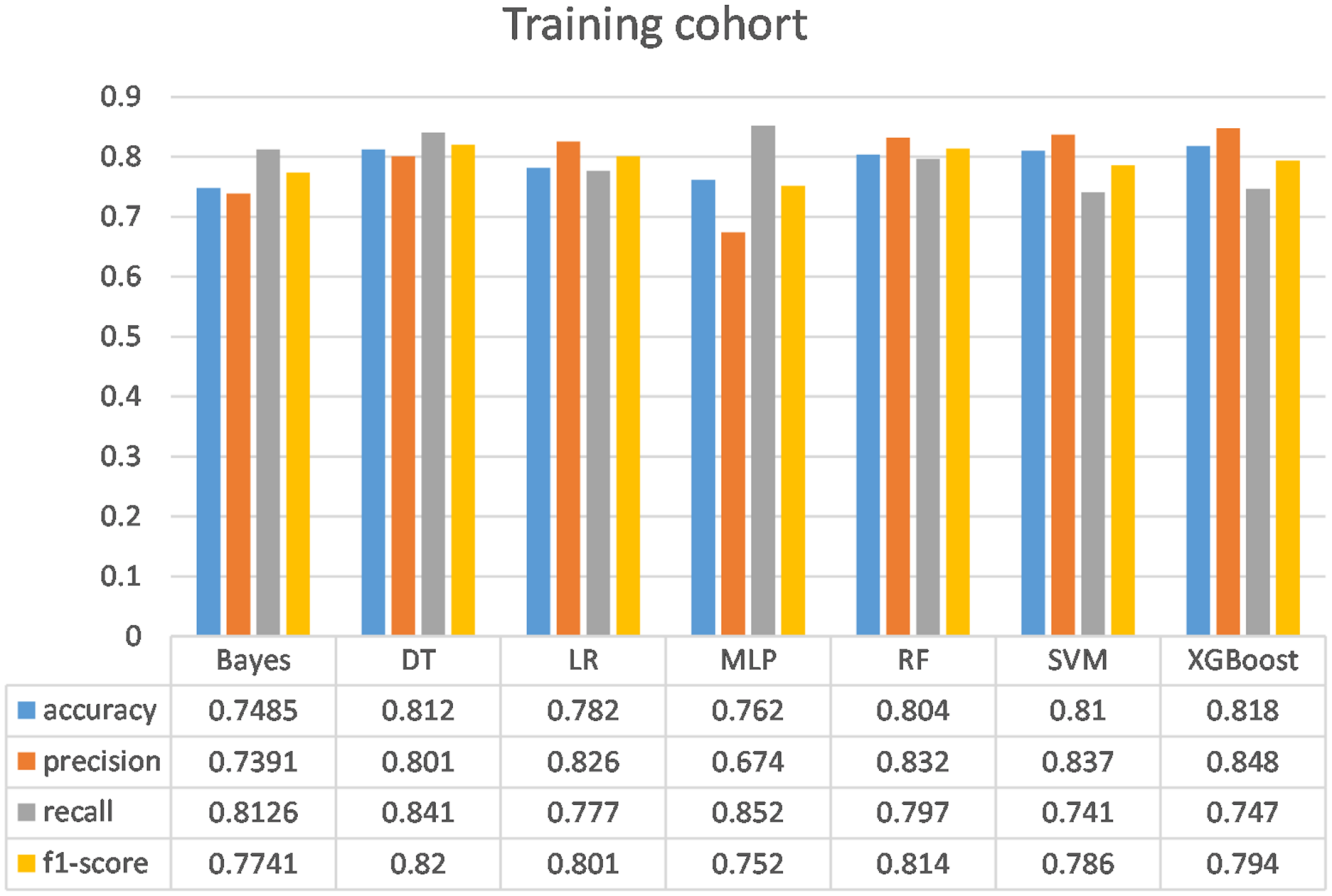
Comparison of the performance of the seven models in the training cohort. Bayes, Bayesian Network; DT, Decision tree; LR, Logistic regression model; MLP, Multilayer perceptron; RF, Random Forest model; SVM, Support vector machine; XGBoost, eXtreme Gradient Boosting.

**Figure 7:**
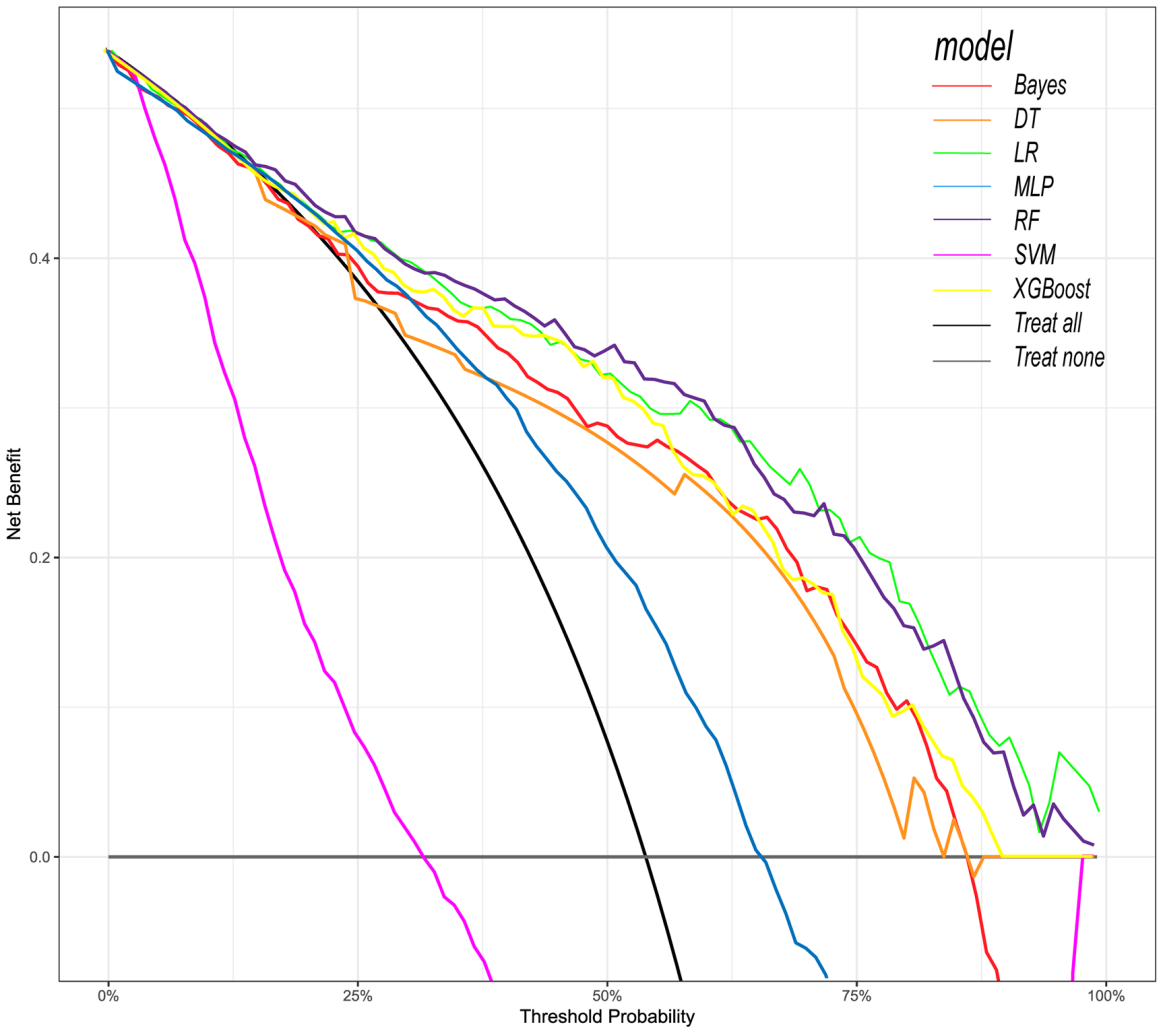
Decision curve analysis (DCA) of seven prediction models. The net benefit curve of the prognostic model was shown. The x-axis represents the threshold probability of intensive care outcome, and the y-axis represents the net benefit. Red line = Bayes model, orange line = DT model, green line = LR model, blue line = MLP model, dark purple line = RF model, bright purple line = SVM model, yellow line = XGBoost model, black line = Treat all, gray line = Treat none.

Following the identification of key predictive factors through rigorous feature selection, we developed and evaluated seven ML models (Bayes, Bayesian Network; DT, Decision Tree; LR, Logistic Regression; MLP, Multilayer Perceptron; RF, Random Forest; SVM, Support Vector Machine; and XGBoost, eXtreme Gradient Boosting) to assess their predictive performance, which demonstrated robust discrimination ability, with the AUC (95% CI) of the training cohort being 0.819 (0.792, 0.845), 0.841 (0.814, 0.865), 0.851 (0.828, 0.875), 0.87 (0.847, 0.892), 0.906 (0.887, 0.924), 0.897 (0.877, 0.916), and 0.904 (0.886, 0.923), respectively (**Figure 4**). The RF algorithm model showed that the training cohort has the highest AUC. The XGBoost model was second only to RF in performance and significantly better than the other five models The AUC (95% CI) of the validation cohort were 0.817 (0.775, 0.859), 0.779 (0.735, 0.822), 0.856 (0.819, 0.893), 0.781 (0.737, 0.825), 0.835 (0.797, 0.873), 0.847 (0.809, 0.885), 0.869 (0.834, 0.904) (**Figure 5**). In the validation cohort, the XGBoost model has the highest AUC, followed by LR, and the RF model has the fourth highest AUC. The results of the accuracy, precision, recall, and F1-score of the seven models were shown in **Figure 6** and **Supplementary Figure S4**. The performance of XGBoost classification model is better than other models. According to the DCA results of the seven prediction models (**Figure 7**), the net benefit of RF is greater than that of other models. In this study, the ROC curve and DCA curve of the training cohort and the validation cohort were evaluated, and the classification ability, calibration degree, and clinical application value of each model were compared. The XGBoost model was the best, although its net income was slightly less than that of RF.

**Figure 8:**
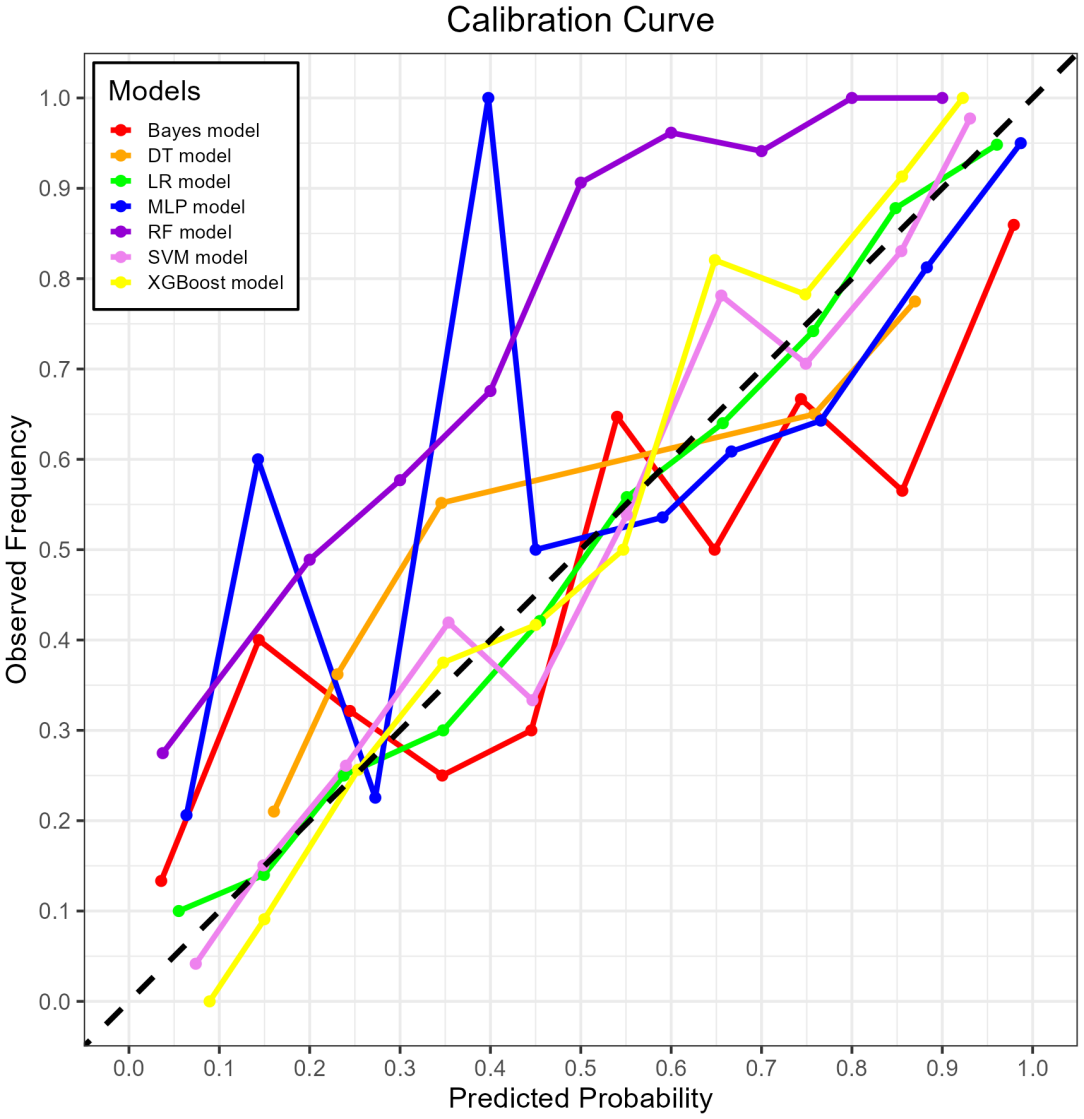
The calibration curve plot of the seven models. Red line = Bayes model, orange line = DT model, green line = LR model, blue line = MLP model, dark purple line = RF model, bright purple line = SVM model, yellow line = XGBoost model.

The XGBoost model demonstrated excellent calibration, with Brier scores of 0.142 (training) and 0.178 (validation)—well below the 0.25 random prediction threshold—indicating high probabilistic precision. The strong calibration performance – evidenced by Brier score 0.143, calibration slope 1.18, and visual alignment in **Figure 8** – confirms that predicted probabilities reliably reflect actual mortality risk.

Both RF and XGBoost demonstrated excellent discrimination in the training cohort (RF AUC=0.906; XGBoost AUC=0.904). However, RF showed poorer generalization in validation (AUC=0.835 vs. XGBoost’s 0.869), indicating potential overfitting from RF’s deep-tree architecture capturing noise in training data. ​​Complementing this, calibration metrics revealed a similar pattern: while RF achieved a Brier score of 0.1816 in validation, XGBoost attained slightly better calibration (Brier=0.1783), confirming its probabilistic reliability.​​ This enhanced stability stems from XGBoost’s ​​regularization mechanisms​​ (L1/L2 penalties, column subsampling), which mitigate overfitting whereas RF lacks comparable constraints. Thus, XGBoost was selected as the optimal model, as its ​​superior validation performance​​ (higher AUC + better Brier score) reflects greater clinical utility for probability-based decisions, despite marginally lower training performance. This addition reinforces our selection rationale through both discrimination and calibration perspectives.

### 3.4 Interpretability analysis

**Figure 9:**
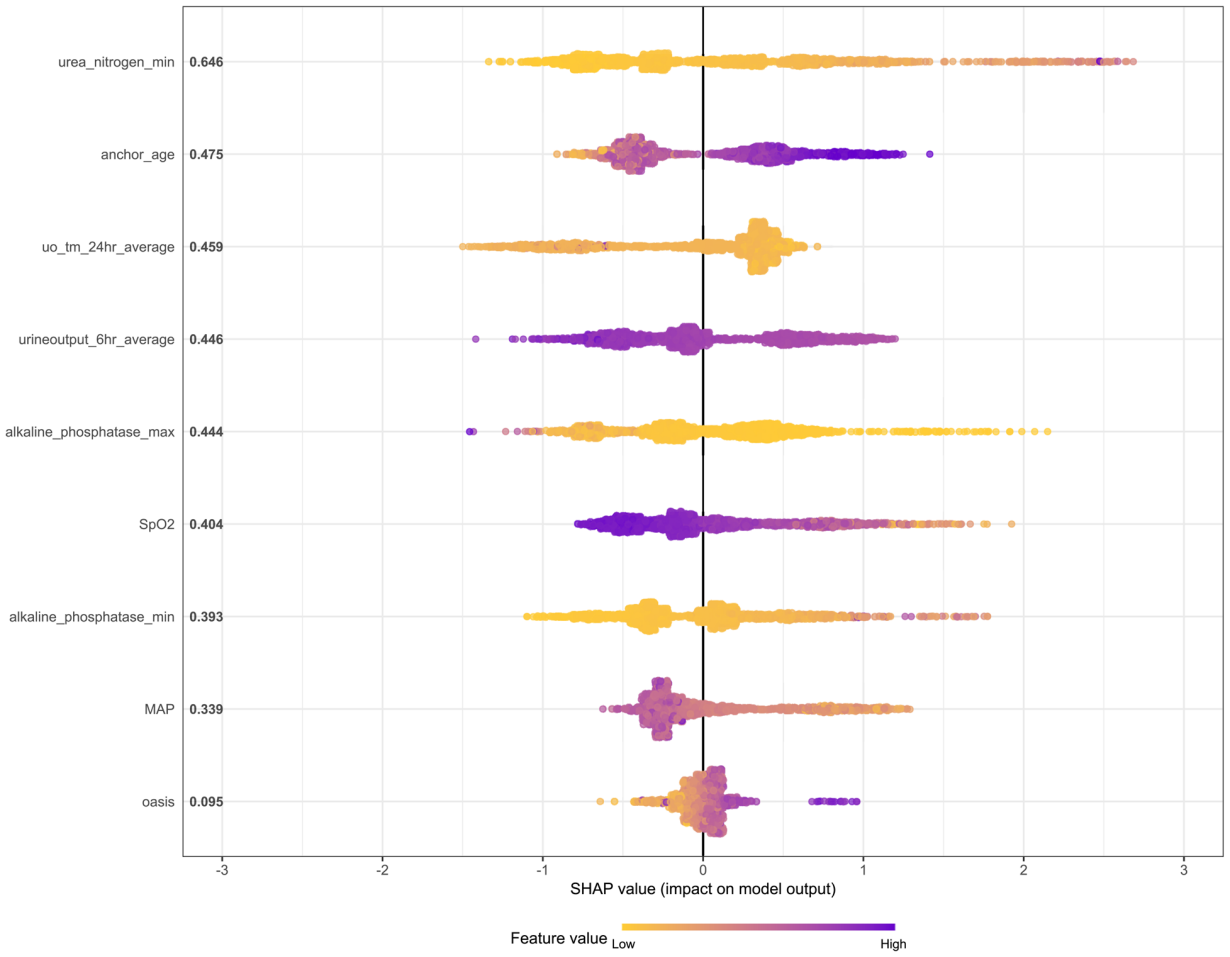
The SHAP method is used to analyze the important features of the XGBoost model. Create a point for each feature attribute value of each patient’s model, thereby assigning a point to each patient on the line for each feature. Dots are colored according to the eigenvalues of the corresponding patients and accumulate vertically to depict the density. Purple indicates high eigenvalues (death in this case), while yellow indicates low eigenvalues. The farther the point is from the baseline SHAP value, the greater the impact on the output.

First, the global interpretability of the baseline model was studied. The XGBoost model was considered the baseline model because it was found to be the best-performing model. Feature importance estimates were based on the overall sample of the training cohort. The global importance of each feature we estimated in SHAP was used to understand the general impact of various features in all samples (**Figure 9**).

The SHAP summary graph illustrates the entire distribution of the impact of each feature on the model output. Color enables us to understand how changes in eigenvalues affect changes in results. Purple represents high eigenvalues, while yellow represents low eigenvalues. The farther a point is from the baseline SHAP value zero, the greater its impact on the output. This allows a better understanding of the relationship between features and the SHAP value (as well as the predicted output). It can be seen from the figure that urea nitrogen min plays a crucial role compared with other risk factors (such as MAP and OASIS).

**Figure 10:**
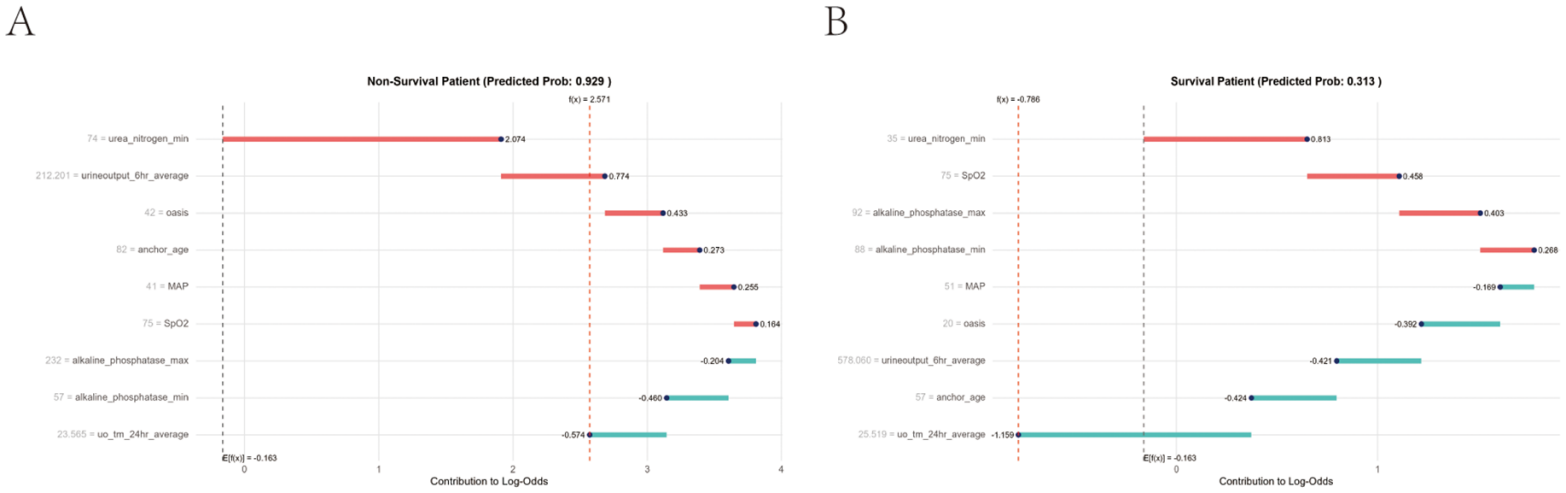
Local model explanation by the Shapley Additive Explanations (SHAP) method. **(A)** Non-survival patient. **(B)** Survival patient. Each patient is represented by the x-axis, while the feature contribution is represented by the y-axis: an increased red part for each individual patient represents a greater probability toward the decision of “Non-survival”.

In addition, local interpretation analyzed the results of specific predictions for individual patients. **Figure 10A** presents data from a urosepsis patient who died during ICU hospitalization. Our prediction model assigned this patient a mortality probability of 96%. The figure demonstrates that urea nitrogen min, urine output 6hr average, OASIS, anchor age, MAP, and SpO_2_ contributed to biasing the prediction towards mortality, whereas alkaline phosphatase max, alkaline phosphatase min, and uo tm 24hour average reduced the predicted risk of death. **Figure 10B** illustrates data from a surviving urosepsis patient during ICU hospitalization, specifically highlighting features favoring mortality and their actual measurements. In this case, the model predicted a 31.3% probability of mortality. The x-axis denotes individual patients, and the y-axis represents feature contributions. For each patient, the extent of the red area indicates the magnitude of contribution towards a ‘non-survival’ prediction.

Beyond interpretability, SHAP results translate into actionable clinical protocols. For patients with critically elevated urea nitrogen levels, intensivists should prioritize dynamic renal function monitoring and initiate early nephroprotective interventions to mitigate acute kidney injury—a dominant predictor of mortality. Similarly, sustained depression of mean arterial pressure (MAP) below clinically significant thresholds warrants immediate hemodynamic optimization, including fluid resuscitation and vasopressor escalation when indicated. These data-driven alerts, derived from SHAP’s quantification of feature contributions, can be integrated into ICU monitoring systems to proactively guide bedside decisions, converting model insights into tangible clinical workflows.

## 4 Discussion

In recent years, ML algorithms have become increasingly popular in the medical field, helping clinicians diagnose diseases faster and more accurately, while achieving personalized treatment plans. In this study, we first used ML methods to construct a predictive model for major adverse prognostic events in patients with urosepsis. It has been proven that ML methods can explain the key characteristics of patients with urosepsis and establish high-precision death prediction models. Compared with traditional risk models, this ML based approach considers the complex interactions between variables and can dynamically adjust based on individual patient characteristics.

Our research analyzed demographic data, vital signs, 80 laboratory indicators, advanced life support data, and comorbidities of patients with urosepsis within 24 hours after admission. Specifically, we use RF, Lasso, Boruta, and XGBoost to model the variables of influencing factors. Then, we rank the variables with non-zero coefficients according to their impact on the outcome variables and identify common variables by taking the intersection of variables selected by all four methods. Finally, 12 common variables were obtained. Combined with systematic review, meta-analysis, and expert clinical opinions, nine factors affecting the outcome were identified as predictors, including urea nitrogen minimum, age, urine output (24-hour average), urine output (6-hour average), alkaline phosphatase maximum, SpO2, alkaline phosphatase minimum, MAP, and OASIS.

Urine output and urea nitrogen are closely related to the main adverse prognostic events of patients (Sun et al., 2022a). Urine output plays an important role in predicting the mortality rate of urosepsis patients. This result has been confirmed in many related studies (Zhang et al., 2021; Kasugai et al., 2023; Yamamoto et al., 2025). Surprisingly, urea nitrogen is the most important predictor of mortality in patients with urosepsis. Urea nitrogen is a metabolic waste produced by the liver, which enters the kidneys through the bloodstream and is then filtered out by the kidneys (Li et al., 2021). Urea nitrogen levels are important indicators of kidney function, water balance, and protein metabolism. Compared to infection and inflammation markers, urea nitrogen is a low-cost, easily accessible indicator that can reflect kidney damage in patients. Previous research results have shown that urosepsis significantly reduces renal blood flow and renal function, thereby further increasing urea nitrogen levels (Tóth-Heyn et al., 2000; Langenberg et al., 2006; Seely et al., 2011). Therefore, urea nitrogen levels can reflect the organ dysfunction status of patients with urosepsis, and severe organ dysfunction is one of the main causes of death in patients (Wang et al., 2024a). However, previous death prediction models for patients with urosepsis did not use this key factor (Mauk, 2018; Gou et al., 2023).

In addition, our research also suggests that alkaline phosphatase (AP) is an important predictor of prognosis in patients with urosepsis. AP is an endogenous detoxifying enzyme that exists throughout the body in four different isoenzymes: germ cell AP, intestinal AP, placental AP, and a non-specific form primarily derived from the kidneys, liver, and bones (Millán, 2006). AP can dephosphorylate endotoxins (Poelstra et al., 1997; Bentala et al., 2002; Verweij et al., 2004). Through its dephosphorylation ability, AP can not only detoxify endotoxins, but also various compounds. The AP measured in serum is currently used as a diagnostic tool for liver disease (Siddique and Kowdley, 2012), bone disease (Ross et al., 2000), and testicular cancer (Neumann et al., 2011). AP may also play an important role in the treatment of critical illnesses. Its dephosphorylation ability can counteract the adverse cascade reactions of molecules such as PAMP (Koyama et al., 2002) and DAMPs (Picher et al., 2003) during urosepsis. Our study found that AP can also serve as a predictor of outcomes in patients with urosepsis. The maximum and minimum values of AP in patients with urosepsis within 24 hours of admission are important predictors of patient prognosis. This discovery may help clinicians evaluate the patient’s condition within 24 hours of admission and determine the patient’s prognosis early.

We used the above nine features to construct ML models to predict the prognosis of patients. Among the ML models, the XGBoost model performs the best. XGBoost is a popular ML algorithm in recent years, characterized by fast computing speed, strong generalization ability, and high predictive performance (Hou et al., 2020; Yue et al., 2022). In the ROC curve, XGBoost performs second only to the RF model in the training cohort, but shows the best discriminative ability in the internal validation cohort. When the threshold probability is between 12.5% and 70%, The clinical intervention guided by the XGBoost model provided greater net benefits in the training cohort. DCA indicates that the RF model has the greatest benefit within a reasonable threshold probability, but considering its poor performance in the validation cohort ROC curve, this means that the RF model may not be optimal. Based on the performance of the model in the training and validation cohorts, the XGBoost model has higher clinical application value and good clinical practicality compared to other models. Finally, we used SHAP values to reveal the ‘black box’ of ML (Molnar, 2019). The SHAP summary diagram illustrates the overall distribution of the impact of each feature on the model output. SHAP is a flexible method that can be used to interpret individual predictions and global interpretations. SHAP tries to provide an intuitive visualization of how different characteristics affect the predicted outcome. One advantage of SHAP for global interpretation is that SHAP reveals not only the importance of features but also their relationship to output. In addition, the prediction of SHAP is reasonably distributed among eigenvalues. These factors are essential to ensure trust in the technology. Our interpretability framework adheres to established principles for explainable clinical AI (Molnar, 2020), transforming complex model predictions into clinically intuitive decision support.

Our findings demonstrate that ML models substantially outperform conventional prognostic tools—both in accuracy and clinical interpretability. Specifically, while traditional methods like logistic regression (LR)—representing linear modeling approaches—achieved competent validation performance (AUC: 0.856), XGBoost surpassed it by a clinically significant margin (ΔAUC: +0.013; **Figure 5**). This gap arises from ML’s ability to capture complex, non-linear interactions that traditional models intrinsically miss. Furthermore, ML uniquely identified dynamic predictors like alkaline phosphatase extrema, whereas conventional methods favor static, guideline-driven variables. Thus, ML transcends incremental accuracy gains; it uncovers pathophysiology-driven decision pathways, converting rigid scores into adaptive, patient-specific prognostication.

However, our research is not without limitations. Firstly, our training cohort and internal validation cohort are both from the MIMIC-IV database, with the majority of patients coming from Western countries; Secondly, we did not conduct a more comprehensive study of the database, which may have led us to overlook some key variables, resulting in potential errors; Thirdly, the retrospective and observational nature of this study may lead to selection bias, which may result in the inclusion of patients who do not fully represent all patients in that category. Therefore, conducting a prospective evaluation is necessary to assess the performance of the model in real-world situations. To further evaluate generalizability, future validation will utilize the eICU Collaborative Research Database—capturing heterogeneous ICU practices across U.S. healthcare systems—to assess model performance using identical endpoints (28-day ACM) and metrics (AUC, sensitivity, specificity, F1-score, Brier score, and DCA-derived net benefit). This will be followed by prospective multi-center testing with local hospital data, maintaining consistent predictor variables, outcome definitions, and performance thresholds to ensure cross-population comparability and clinical utility quantification.

## 5 Conclusions

In conclusion, the ML method is a reliable tool for predicting the prognosis of patients with urosepsis. Combining global and local interpretability methods to interpret the intrinsic information from the XGBoost model may prove clinically useful and help clinicians customize precise management to maximize the survival of patients with urosepsis.

## Data Availability

Publicly available datasets were analyzed in this study. This data can be found here: This study utilized publicly available datasets, which can be accessed at https://mimic.mit.edu/.

## Supplementary material

Supplementary Figure 1 Raw data and Mice filled data density plot. Blue line = raw data, red line = interpolated data.

Supplementary Figure 2 LASSO regression path showing the coefficients of variables across different values of the regularization parameter (λ).

Supplementary Figure 3 Cross-validation error plot for selecting the optimal λ in LASSO. The vertical dashed line represents the optimal λ where the minimal cross-validation error is achieved.

Supplementary Figure 4 Comparison of the performance of the seven models in the validation cohort. Bayes, Bayesian Network; DT, Decision tree; LR, Logistic regression model; MLP, Multilayer perceptron; RF, Random Forest model; SVM, Support vector machine; XGBoost, eXtreme Gradient Boosting.
