# Supplementary figures and images for: Significant adverse prognostic events in patients with urosepsis: a machine learning based model development and validation study
Source: Front Cell Infect Microbiol. 2025 Aug 8;15:1623109. doi: 10.3389/fcimb.2025.1623109 (PMC12370708; doi:10.3389/fcimb.2025.1623109)

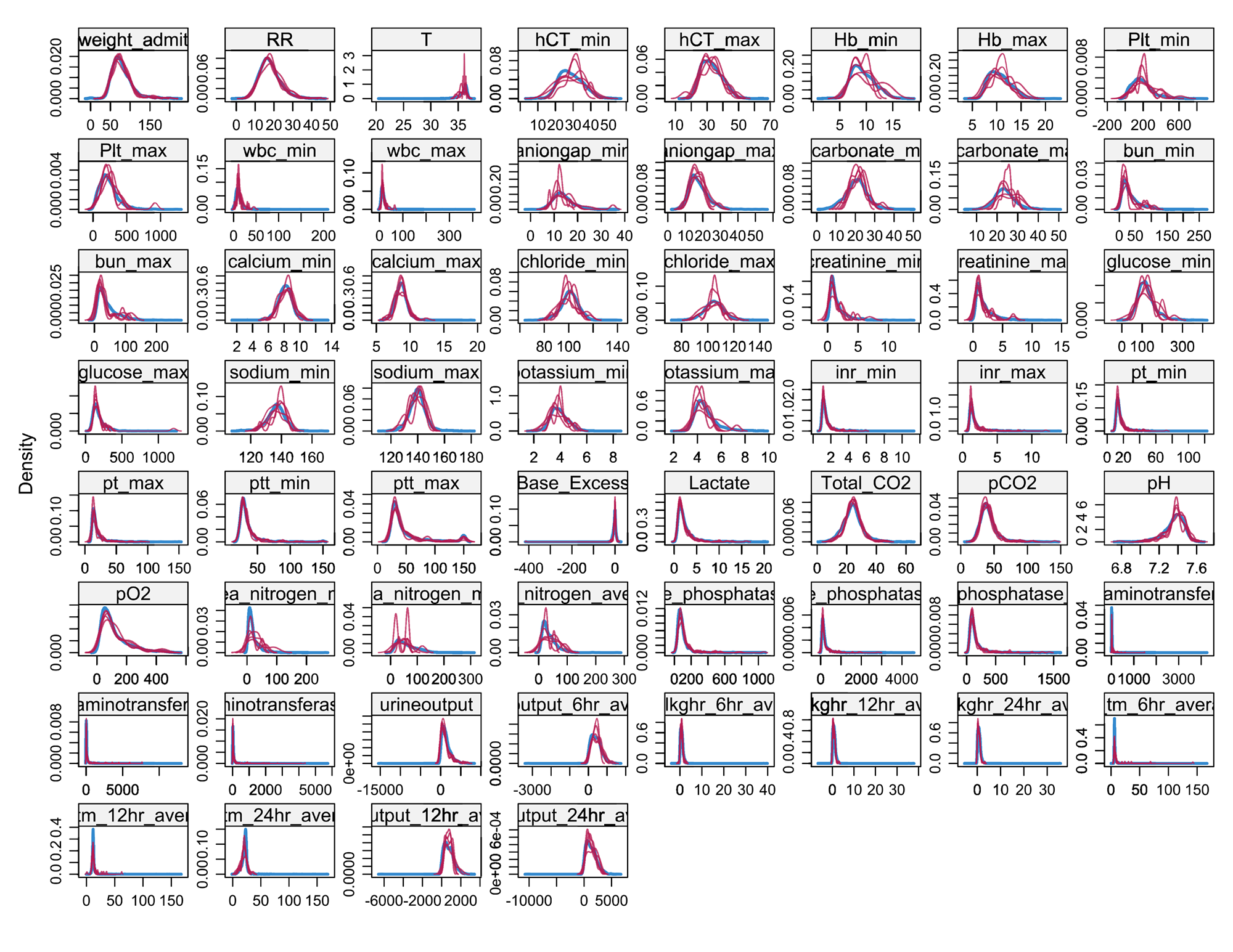

Supplement: Supplementary Figure 1 — Raw data and Mice filled data density plot. Blue line = raw data, red line = interpolated data. [file Image1.tif]

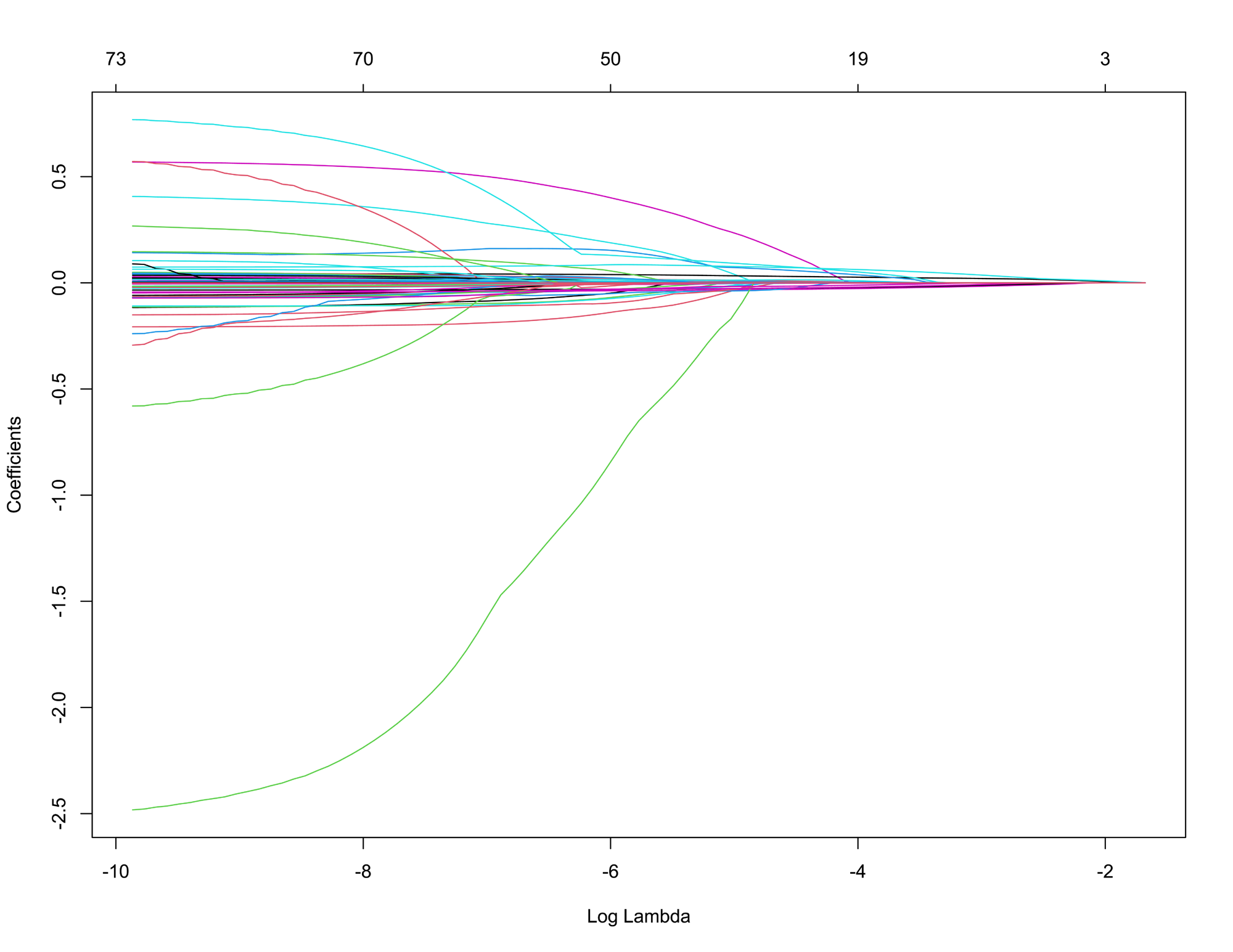

Supplement: Supplementary Figure 2 — LASSO regression path showing the coefficients of variables across different values of the regularization parameter (λ). [file Image2.tif]

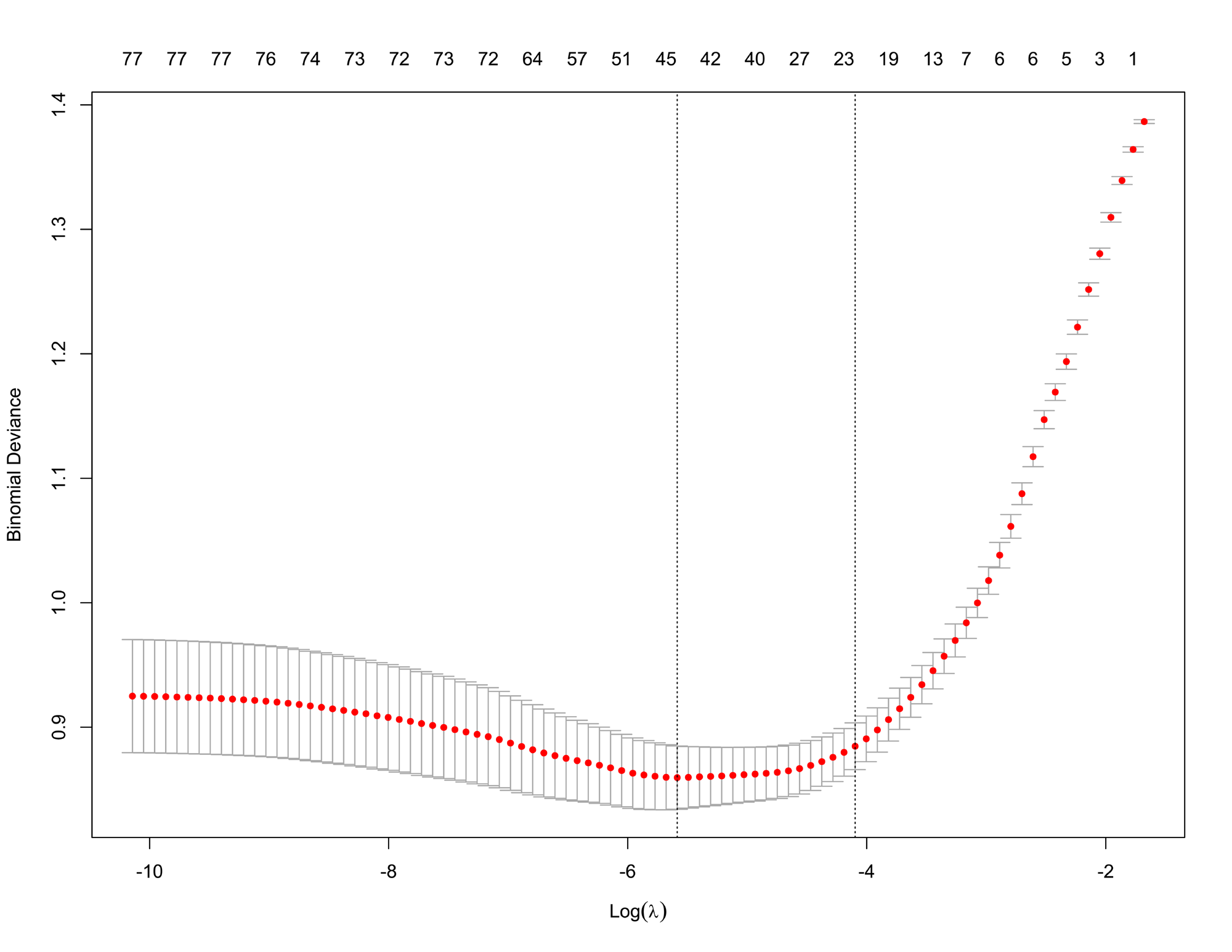

Supplement: Supplementary Figure 3 — Cross-validation error plot for selecting the optimal λ in LASSO. The vertical dashed line represents the optimal λ where the minimal cross-validation error is achieved. [file Image3.tif]

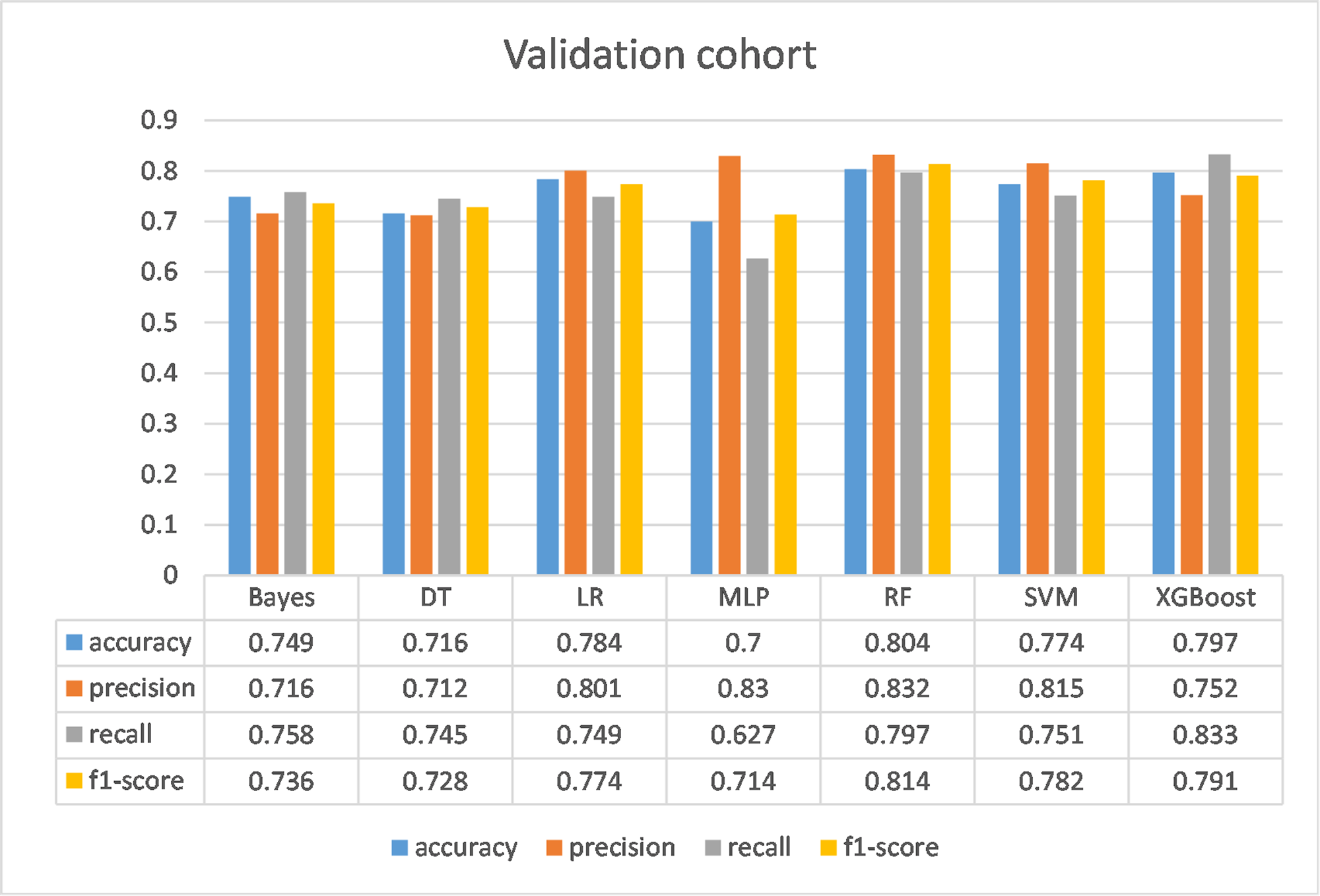

Supplement: Supplementary Figure 4 — Comparison of the performance of the seven models in the validation cohort. Bayes, Bayesian Network; DT, Decision tree; LR, Logistic regression model; MLP, Multilayer perceptron; RF, Random Forest model; SVM, Support vector machine; XGBoost, eXtreme Gradient Boosting. [file Image4.tif]
