# Supplementary material for: Significant adverse prognostic events in patients with urosepsis: a machine learning based model development and validation study
Source: Front Cell Infect Microbiol. 2025 Aug 8;15:1623109. doi: 10.3389/fcimb.2025.1623109 (PMC12370708; doi:10.3389/fcimb.2025.1623109)
Supplement: Supplementary file 5 [file Table1.docx]

**Supplementary Table S1. Patient demographics and baseline characteristics.**

|  | **Training cohort** | | | **Internal validation cohort** | | |
| --- | --- | --- | --- | --- | --- | --- |
| Status | Survivors (n=523) | Non-survivors (n=463) | P-value | Survivors (n=186) | Non-survivors (n=217) | P-value |
| Age | 63.744 ± 14.022 | 73.140 ± 13.580 | <0.001 | 64.113 ± 13.594 | 74.313 ± 12.938 | <0.001 |
| Weight | 79.057 ± 26.467 | 75.943 ± 25.084 | 0.018 | 81.437 ± 22.636 | 80.441 ± 26.549 | 0.294 |
| Gender |  |  | 0.281 |  |  | 0.902 |
| Male | 221 (42.256%) | 180 (38.877%) |  | 86 (46.237%) | 99 (45.622%) |  |
| Female | 302 (57.744%) | 283 (61.123%) |  | 100 (53.763%) | 118 (54.378%) |  |
| Race |  |  | 0.033 |  |  | 0.228 |
| Black | 76 (14.532%) | 48 (10.367%) |  | 26 (13.978%) | 18 (8.295%) |  |
| White | 321 (61.377%) | 315 (68.035%) |  | 113 (60.753%) | 148 (68.203%) |  |
| Yellow | 23 (4.398%) | 10 (2.160%) |  | 3 (1.613%) | 5 (2.304%) |  |
| Others | 103 (19.694%) | 90 (19.438%) |  | 44 (23.656%) | 46 (21.198%) |  |
| Admission_type |  |  | <0.001 |  |  | 0.003 |
| Elective | 149 (28.489%) | 65 (14.039%) |  | 51 (27.419%) | 35 (16.129%) |  |
| Emergency | 283 (54.111%) | 312 (67.387%) |  | 92 (49.462%) | 142 (65.438%) |  |
| Urgent | 91 (17.400%) | 86 (18.575%) |  | 43 (23.118%) | 40 (18.433%) |  |
| Comorbidity(%) |  |  |  |  |  |  |
| Myocardial_infarct | 81 (15.488%) | 94 (20.302%) | 0.048 | 38 (20.430%) | 48 (22.120%) | 0.680 |
| Heart_failure | 166 (31.740%) | 189 (40.821%) | 0.003 | 67 (36.022%) | 99 (45.622%) | 0.051 |
| Hypertension | 265 (50.669%) | 216 (46.652%) | 0.208 | 89 (47.849%) | 88 (40.553%) | 0.141 |
| Diabetes | 207 (39.579%) | 175 (37.797%) | 0.566 | 78 (41.935%) | 82 (37.788%) | 0.396 |
| Acute_Kidney_Failure | 319 (60.994%) | 296 (63.931%) | 0.342 | 103 (55.376%) | 145 (66.820%) | 0.019 |
| Vital signs |  |  |  |  |  |  |
| HR | 61.975 ± 14.710 | 60.136 ± 18.807 | 0.236 | 61.946 ± 15.080 | 62.410 ± 18.697 | 0.352 |
| RR | 17.800 ± 5.264 | 17.680 ± 5.898 | 0.391 | 17.556 ± 5.132 | 18.124 ± 5.833 | 0.262 |
| T | 35.919 ± 0.981 | 35.784 ± 1.114 | 0.003 | 35.909 ± 0.758 | 35.735 ± 0.991 | 0.19 |
| SPO2 | 85.841 ± 9.556 | 78.294 ± 16.763 | <0.001 | 84.339 ± 13.866 | 78.373 ± 14.311 | <0.001 |
| MAP | 54.771 ± 12.635 | 49.045 ± 13.878 | <0.001 | 56.559 ± 11.700 | 46.192 ± 13.372 | <0.001 |
| Laboratory parameters |  |  |  |  |  |  |
| Hct_min | 28.193 ± 6.675 | 29.564 ± 6.594 | 0.002 | 28.169 ± 6.923 | 29.140 ± 6.477 | 0.104 |
| Hct_max | 32.593 ± 6.195 | 33.368 ± 6.399 | 0.035 | 32.809 ± 7.076 | 33.443 ± 6.792 | 0.227 |
| Hb_min | 9.166 ± 2.197 | 9.503 ± 2.166 | 0.022 | 9.133 ± 2.350 | 9.405 ± 2.149 | 0.117 |
| Hb_max | 10.563 ± 2.088 | 10.704 ± 2.119 | 0.248 | 10.586 ± 2.385 | 10.770 ± 2.287 | 0.244 |
| Plt_min | 196.489 ± 123.020 | 180.572 ± 108.093 | 0.049 | 194.054 ± 114.907 | 194.005 ± 120.190 | 0.912 |
| Plt_max | 240.470 ± 145.847 | 215.533 ± 122.555 | 0.004 | 235.882 ± 130.936 | 233.198 ± 134.338 | 0.766 |
| Wbc_min | 11.108 ± 7.741 | 13.146 ± 14.259 | 0.006 | 10.910 ± 6.814 | 12.786 ± 7.276 | 0.002 |
| Wbc_max | 16.575 ± 24.197 | 17.283 ± 17.250 | 0.046 | 14.651 ± 8.567 | 17.174 ± 10.099 | 0.008 |
| Aniongap_min | 13.057 ± 3.717 | 14.419 ± 4.518 | <0.001 | 12.876 ± 3.720 | 14.530 ± 4.238 | <0.001 |
| Aniongap_max | 16.614 ± 5.143 | 18.313 ± 5.593 | <0.001 | 16.742 ± 5.908 | 18.544 ± 5.525 | <0.001 |
| Bicarbonate_min | 21.147 ± 5.462 | 19.946 ± 6.089 | 0.001 | 21.027 ± 5.532 | 20.212 ± 5.694 | 0.038 |
| Bicarbonate_max | 24.050 ± 4.805 | 23.317 ± 5.532 | 0.014 | 24.027 ± 4.523 | 23.627 ± 5.317 | 0.226 |
| Bun_min | 32.046 ± 26.055 | 38.184 ± 27.571 | <0.001 | 32.452 ± 28.146 | 40.309 ± 31.918 | <0.001 |
| Bun_max | 37.780 ± 29.955 | 44.283 ± 31.676 | <0.001 | 38.457 ± 34.372 | 47.032 ± 35.116 | <0.001 |
| Calcium_min | 8.154 ± 0.942 | 8.061 ± 0.978 | 0.471 | 8.168 ± 0.882 | 8.037 ± 0.886 | 0.116 |
| Calcium_max | 8.760 ± 1.007 | 8.655 ± 0.912 | 0.204 | 8.670 ± 1.031 | 8.745 ± 1.030 | 0.431 |
| Chloride_min | 100.650 ± 7.200 | 101.123 ± 7.770 | 0.485 | 101.011 ± 7.681 | 99.484 ± 8.186 | 0.044 |
| Chloride_max | 104.738 ± 7.389 | 105.374 ± 8.144 | 0.219 | 104.935 ± 7.859 | 104.175 ± 8.377 | 0.443 |
| Creatinine_min | 1.464 ± 1.320 | 1.635 ± 1.323 | <0.001 | 1.572 ± 1.456 | 1.862 ± 1.664 | 0.002 |
| Creatinine_max | 1.777 ± 1.595 | 1.957 ± 1.574 | <0.001 | 1.920 ± 1.873 | 2.250 ± 1.891 | <0.001 |
| Glucose_min | 124.642 ± 44.219 | 119.678 ± 52.843 | 0.006 | 119.656 ± 38.569 | 122.295 ± 41.809 | 0.51 |
| Glucose_max | 183.201 ± 100.606 | 180.676 ± 99.014 | 0.566 | 178.435 ± 77.941 | 186.180 ± 123.015 | 0.745 |
| Sodium_min | 136.518 ± 5.682 | 136.829 ± 6.434 | 0.354 | 136.710 ± 6.726 | 135.604 ± 6.484 | 0.091 |
| Sodium_max | 139.859 ± 5.941 | 140.410 ± 6.729 | 0.288 | 140.113 ± 6.997 | 139.217 ± 6.104 | 0.207 |
| Potassium_min | 3.875 ± 0.629 | 3.965 ± 0.695 | 0.097 | 3.875 ± 0.585 | 4.037 ± 0.648 | 0.007 |
| Potassium_max | 4.587 ± 0.847 | 4.727 ± 0.974 | 0.041 | 4.637 ± 0.938 | 4.841 ± 0.931 | 0.01 |
| INR_min | 1.414 ± 0.608 | 1.638 ± 0.959 | <0.001 | 1.445 ± 0.639 | 1.620 ± 0.810 | 0.029 |
| INR_max | 1.624 ± 0.870 | 2.047 ± 1.663 | <0.001 | 1.676 ± 1.110 | 1.956 ± 1.316 | 0.019 |
| Pt_min | 15.514 ± 6.756 | 17.908 ± 10.293 | <0.001 | 15.678 ± 6.551 | 17.541 ± 8.015 | 0.012 |
| Pt_max | 17.888 ± 9.442 | 21.714 ± 16.447 | <0.001 | 17.952 ± 9.413 | 21.094 ± 13.859 | 0.024 |
| Ptt_min | 32.709 ± 13.302 | 36.457 ± 18.048 | <0.001 | 34.161 ± 15.127 | 34.573 ± 13.168 | 0.13 |
| Ptt_max | 46.250 ± 31.684 | 52.518 ± 36.071 | <0.001 | 47.825 ± 33.519 | 51.884 ± 35.499 | 0.026 |
| Base_excess | -1.155 ± 5.353 | -3.186 ± 20.100 | 0.002 | -1.290 ± 6.522 | -2.507 ± 6.765 | 0.011 |
| Lactate | 2.120 ± 1.486 | 2.495 ± 1.955 | <0.001 | 2.396 ± 2.591 | 2.683 ± 2.173 | <0.001 |
| Total_CO2 | 24.384 ± 5.862 | 23.793 ± 6.857 | 0.051 | 24.500 ± 6.283 | 23.737 ± 6.877 | 0.062 |
| PH | 7.377 ± 0.094 | 7.351 ± 0.111 | <0.001 | 7.366 ± 0.109 | 7.354 ± 0.119 | 0.207 |
| PCO2 | 41.197 ± 12.371 | 41.931 ± 14.135 | 0.67 | 42.495 ± 14.202 | 41.244 ± 14.841 | 0.121 |
| PO2 | 130.818 ± 98.314 | 105.687 ± 82.842 | <0.001 | 125.226 ± 99.754 | 112.728 ± 91.904 | 0.218 |
| Urea_Nitrogen_min | 13.685 ± 10.898 | 29.153 ± 24.353 | <0.001 | 13.677 ± 10.387 | 30.323 ± 28.933 | <0.001 |
| Urea_Nitrogen_max | 62.979 ± 39.177 | 56.542 ± 35.001 | 0.031 | 62.306 ± 36.845 | 63.046 ± 40.518 | 0.962 |
| Urea_Nitrogen_average | 32.531 ± 20.129 | 41.342 ± 27.996 | <0.001 | 32.183 ± 18.499 | 44.163 ± 31.390 | <0.001 |
| Alkaline_Phosphatase_min | 88.163 ± 76.000 | 119.624 ± 122.128 | <0.001 | 77.946 ± 34.841 | 104.908 ± 68.642 | <0.001 |
| Alkaline_Phosphatase_max | 285.709 ± 410.723 | 195.883 ± 203.912 | <0.001 | 207.806 ± 247.429 | 189.880 ± 202.895 | 0.195 |
| Alkaline_Phosphatase_average | 163.916 ± 169.847 | 149.007 ± 137.006 | 0.023 | 132.005 ± 111.046 | 141.573 ± 103.136 | 0.359 |
| Alanine_Aminotransferase_min | 17.191 ± 20.485 | 59.985 ± 311.719 | <0.001 | 16.640 ± 15.286 | 54.258 ± 149.680 | <0.001 |
| Alanine_Aminotransferase_max | 241.671 ± 785.162 | 265.067 ± 1001.353 | 0.003 | 175.097 ± 388.552 | 266.300 ± 731.372 | 0.751 |
| Alanine_Aminotransferase_average | 65.722 ± 122.653 | 148.282 ± 541.367 | 0.826 | 56.881 ± 85.233 | 141.761 ± 400.187 | 0.204 |
| Urineoutput | 1560.851 ± 1242.168 | 1184.795 ± 1064.437 | <0.001 | 1516.935 ± 1099.153 | 1123.817 ± 1461.666 | 0.001 |
| Urineoutput_6hr_average | 492.212 ± 264.715 | 325.017 ± 231.804 | <0.001 | 493.716 ± 265.389 | 306.622 ± 327.890 | <0.001 |
| Urineoutput_12hr_average | 893.198 ± 511.387 | 590.334 ± 428.442 | <0.001 | 888.771 ± 505.461 | 559.708 ± 624.914 | <0.001 |
| Urineoutput_24hr_average | 1593.158 ± 978.279 | 1042.014 ± 790.618 | <0.001 | 1601.262 ± 986.139 | 994.304 ± 1078.292 | <0.001 |
| Urineoutput_mlkghr_6hr_average | 1.062 ± 1.807 | 0.663 ± 0.528 | <0.001 | 0.910 ± 0.586 | 0.805 ± 2.610 | <0.001 |
| Urineoutput_mlkghr_12hr_average | 1.042 ± 1.721 | 0.656 ± 0.513 | <0.001 | 0.902 ± 0.571 | 0.761 ± 2.463 | <0.001 |
| Urineoutput_mlkghr_24hr_average | 0.993 ± 1.020 | 0.649 ± 0.503 | <0.001 | 0.906 ± 0.577 | 0.763 ± 2.443 | <0.001 |
| Urineoutput_tm_6hr_average | 8.706 ± 11.340 | 7.409 ± 7.709 | <0.001 | 8.371 ± 7.818 | 6.876 ± 2.908 | 0.002 |
| Urineoutput_tm_12hr_average | 14.115 ± 11.615 | 12.400 ± 7.690 | <0.001 | 13.475 ± 7.240 | 11.790 ± 2.888 | <0.001 |
| Urineoutput_tm_24hr_average | 23.576 ± 12.818 | 20.667 ± 8.593 | <0.001 | 22.482 ± 7.054 | 19.948 ± 4.532 | <0.001 |
| Advanced life support |  |  |  |  |  |  |
| Mechvent | 237 (45.315%) | 220 (47.516%) | 0.489 | 72 (38.710%) | 106 (48.848%) | 0.041 |
| Sore |  |  |  |  |  |  |
| SAPSII_max | 43.723 ± 14.067 | 49.786 ± 13.475 | <0.001 | 42.005 ± 14.022 | 51.945 ± 13.862 | <0.001 |
| OASIS | 34.386 ± 8.664 | 37.844 ± 8.505 | <0.001 | 34.140 ± 8.394 | 39.355 ± 8.546 | <0.001 |
| LODS_max | 6.017 ± 3.168 | 6.940 ± 3.017 | <0.001 | 5.731 ± 3.154 | 7.392 ± 3.218 | <0.001 |

Categorical data were showed as frequency (percentage). Continuous variables with normal distributions were presented as the mean (SD, standard deviation) and compared with independent samples t tests. Non-normally distributed variables are expressed as the median (interquartile ranges), which were compared with Kruskal-Wallis test.
