# Supplementary material for: Significant adverse prognostic events in patients with urosepsis: a machine learning based model development and validation study
Source: Front Cell Infect Microbiol. 2025 Aug 8;15:1623109. doi: 10.3389/fcimb.2025.1623109 (PMC12370708; doi:10.3389/fcimb.2025.1623109)
Supplement: Supplementary file 6 [file Table2.docx]

**Table S2. Standardized Terminology and Abbreviations Glossary.**

| **Abbreviation** | **Standardized Term** |
| --- | --- |
| ABG | arterial blood gas |
| ACM | All-Cause Mortality |
| AUC | area under the ROC curve |
| Bayes | Bayesian Network |
| DCA | Decision Curve Analysis |
| DNN | Deep Neural Networks |
| DT | Decision Tree |
| GBDT | Gradient Boosting Decision Trees |
| HR | heart rate |
| IRB | institutional review board |
| IRP | intrarenal pressure |
| LR | Logistic Regression |
| MAP | mean arterial pressure |
| MIMIC-IV | Medical Information Mart for Intensive Care IV |
| MIT | Massachusetts Institute of Technology |
| ML | machine learning |
| MLP | Multilayer Perceptron |
| PCT | procalcitonin |
| PLR | Platelet-to-Lymphocyte Ratio |
| RF | Random Forest |
| RR | respiratory rate |
| SHAP | SHapley Additive exPlans |
| SOFA | Sequential Organ Failure Assessment |
| SpO2 | saturation of peripheral oxygen |
| SVM | Support Vector Machine |
| T | body temperature |
| URS | ureteroscopy |
| UTI | urinary tract infections |
| XGBoost | eXtreme Gradient Boosting |

Reference glossary for standardized terminology and abbreviation conventions used throughout the manuscript. All clinical variables, model names, and technical terms adhere to these designations.
